# Cardiovascular and metabolic risk factors of shift workers within the automotive industry

**DOI:** 10.4102/hsag.v24i0.1227

**Published:** 2019-10-14

**Authors:** Andre L. Travill, Farzaanah Soeker, Dillon Overmeyer, Frederic Rickers

**Affiliations:** 1 Department Sport, Recreation and Exercise Science, Faculty of Community and Health Sciences, University of the Western Cape, Bellville, South Africa

**Keywords:** cardiovascular disease, metabolic, risk factors, cholesterol, glucose, automotive industry, shift workers

## Abstract

**Background:**

Previous research highlighted the importance of identifying the modifiable risk factors among shift workers in specific industries to take effective preventative and therapeutic steps to decrease the risks associated with non-communicable chronic diseases.

**Aim:**

The aim of this study was to investigate the prevalence of cardiovascular and metabolic disease risk factors among shift workers within the automotive industry.

**Setting:**

This study was conducted at a car manufacturing company in the Eastern Cape Province of South Africa.

**Methods:**

The study employed a cross-sectional quantitative design. Body mass index was calculated, and the American College for Sports Medicine classification for normal weight, overweight and obesity was used to assess the weight status of the workers. Fasting blood glucose and cholesterol as well as blood pressure (BP) were also measured.

**Results:**

Seventy-five automotive shift workers participated in the research. Twenty-three per cent of the participants had no risk factors, 30.6% had one risk factor, 34.7% had two risk factors and only 5.3% exhibited four risk factors. Sixty-six percent of participants were classified as either pre-obese or obese, while 55% were hypertensive. Systolic BP (*r* = 0.258; *p* < 0.05), diastolic BP (*r* = 0.342; *p* < 0.01), cholesterol (*r* = 0.258; *p* < 0.05) and age (*r* = 0.271; *p* < 0.05) significantly correlated with body mass index.

**Conclusion:**

This study highlights the prevalence of risk factors for cardiovascular diseases among employees in the automotive industry. However, it does not show any risk factors for metabolic diseases.

## Introduction

According to the World Health Organization (2017), 15 million people die annually because of non-communicable diseases (NCDs). Non-communicable diseases are already among the top causes of death in South Africa, with cardiovascular diseases (CVDs) the leading cause in this category (Nojilana et al. 2016; World Health Organization 2017). Hypertension, hypocholesteraemia, diabetes mellitus, obesity and inactive lifestyles are some of the key contributing factors for CVDs (World Health Organization 2017).

Because workers spend a significant part of their lives in the workplace, it is reasonable to assume that the work environment will either advance or counter the impact of the determinants of CVDs. Physical activity, which is considered a major role-player in the fight against the global health issues of obesity and high blood pressure (BP) (Köchli et al. 2018; Koolhaas et al. 2017), has been drastically reduced in most industries by the introduction of labour-saving equipment and devices brought about by rapid technological advances (Esquirol et al. 2011). Furthermore, competition between manufacturers and production sites has led to extended working hours that are generally organised into shifts (Lehndorff n.d.). Shift work is defined as work that takes place outside conventional daytime hours, and it can include early morning, evening and night work as well as rotating shifts (Kivimäki, Batty & Hublin 2011). Various researchers have linked shift work, especially night shift work, to increase in weight (Marquezea et al. 2012; Puttonen, Harma & Hublin 2010 ) and a heightened risk of developing CVDs (Vyas et al. 2012) and metabolic diseases. Marquezea et al. (2012) found weight gain during night work is more pronounced than weight gain during daytime work among nurses. Vetter et al. (2016) reported that a longer duration of rotating night shift work among registered nurses was associated with an absolute increase in the risk for coronary heart disease. Disrupted circadian cycles are considered the key contributor to these negative health effects of shift work (Potter et al. 2016). To assess the cardiovascular and metabolic risk factors of the participants, the following variables were evaluated in the current study: blood glucose, cholesterol, BP, height, weight and body mass index (BMI).

### Problem statement

Despite the significant impact of chronic diseases on productivity in the workplace, there is a dearth in scientific information on the prevalence of cardiovascular and metabolic risk factors in most industries, but more specifically among shift workers in the automotive industry in South Africa. The objective of this study was to determine the cardiovascular and metabolic risk factors in automotive industry shift workers.

### Significance of the study

The health of workers has significant consequences on the productivity in the workplace. The extent of the prevalence of NCDs is not fully known and understood especially in the South African automotive industry. The results of this study will reveal the extent of the problem, and the data can be used by both employers and employees to minimise risks in the future.

## Research design and method

### Design

The study employed a cross-sectional quantitative design, as it examined the risk profiles of the automotive shift workers at a specific point in time.

### Population and sampling

Data for this project were extracted from an established database of automotive workers, which were collected by qualified Biokineticists. Participants had to complete a wellness screening questionnaire after consenting to be tested. The form included a section where participants gave consent for their data to be used for research purposes and to compile their risk profiles. The researchers of the current project were responsible for the extraction and analysis of the collected data. Ethics clearance for the research was obtained from the university under whose authority the research was conducted (registration number 15/4/116). The data of 75 randomly selected shift workers (54 females and 21 males) were extracted from the automotive industry database. The following variables were selected to assess the cardiovascular and metabolic risk factors: blood glucose, cholesterol, BP, height, weight and BMI.

### Blood glucose and cholesterol

Fasting blood glucose and cholesterol were assessed using a portable glucometer (Accutrend) and compatible blood glucose and cholesterol strips. Fasting blood glucose and cholesterol were categorised according to the American College of Sport Medicine’s guidelines for exercise testing and prescription (Pescatello 2014). Values above 6.2 mmol/L for glucose and 5.18 mmol/L for cholesterol were considered metabolic risk factors.

### Blood pressure

Blood pressure was measured using a sphygmomanometer and stethoscope. The procedure was performed while the participant was in a sitting position with the arm at the level of the heart and after a minimum rest of 5 min. The measurements were recorded in millimetre mercury (mmHg). Participants were classified as hypertensive and at risk if their systolic BP (SBP) was greater or equal to140 mmHg or if their diastolic BP (DBP) was greater than or equal to 90 mmHg (Pescatello 2014).

### Height and weight

Height and weight were measured in accordance with the standard procedures of the International Society for the Advancement of Kinanthropometry (2011) to have Technical Errors of Measurement that are within acceptable standards (< 2%).

The data analyses options for this study were restricted by the extent of the information contained in the database from which the data were extracted. Although the diagnostic accuracy of BMI is limited (Romero-Corral et al. 2008), it was used in the current study to assess the levels of overweight and obesity of the participants. Body mass index was calculated for each subject using the following formula: weight (kg)/[height (m)]^2^. The American College of Sport Medicine (Pescatello 2014) classification for normal weight (BMI 18.5 kg/m^2^ – 24.9 kg/m^2^), overweight (BMI between 25 kg/m^2^ and 29.9 kg/m^2^) and obesity (BMI ≥ 30 kg/m^2^) was used to assess the weight status of participants.

### Data analysis

A descriptive analysis was performed using the Statistical Package for the Social Sciences (SPSS) (V22). Results were either expressed as means and standard deviations or absolute numbers and percentages. An independent samples *t*-test was used to compare the risk profiles of women with that of men. Pearson’s product moment correlation coefficients were performed to determine possible associations between BMI, SBP, DBP, glucose, cholesterol and age. The level of significance was set at *p* < 0.05.

### Ethical consideration

The Senate Research Committee of the University of the Western Cape approved the methods and ethics of the research (Reg. No 15/4/16).

## Results

Demographic and descriptive statistics for all the participants are presented in Table 1. Sixteen per cent of the workers fell in the ≥ 45 years age group. The rest of the participants (84%) were younger than 45 years. All variables that were included for analysis related positively and significantly with BMI except for glucose (Table 2).

**TABLE 1:** Demographic and descriptive statistics for the full group, participants younger than 45 years, participants aged 45 years and older men and women.

Variable	Full group		< 45 years		≥ 45 years		Men		Women	
	Mean	SD	Mean	SD	Mean	SD	Mean	SD	Mean	SD
Age	34.4	10.8	36.3	11.6	53.5	4.8	29.6	6.7	36.4	11.6
BMI	27.9	6.2	27.76	6.5	29.7	4.7	28.3	5.6	27.8	6.5
SBP	120.4	14.1	119.3	13.0	121.3	9.8	123.5	16.3	119.2	13.1
DBP	78.5	10.7	77.91	9.56	80.6	11.7	79.9	13.4	77.6	9.6
Cholesterol	4.5	0.9	4.60	0.90	5.1	0.8	4.11	0.9	4.6	0.9
Glucose	5.9	1.8	5.91	1.97	6.9	2.7	6.10	1.2	5.9	1.9

BMI, body mass index; SBP, systolic blood pressure; DBP, diastolic blood pressure; SD, standard deviation.

**TABLE 2:** Correlation between body mass index, blood pressure, cholesterol, blood glucose and age.

Variable	BMI
Systolic blood pressure	0.258*
Diastolic blood pressure	0.342**
Glucose	0.225
Cholesterol	0.258*
Age	0.271*

BMI, body mass index.

* *p* < 0.05;

** *p* < 0.01.

Sixty-four per cent of the participants were either overweight or obese and 36% were normal weight, while 56% were hypertensive or pre-hypertensive in terms of SBP and 50% in terms of DPB (Table 3). Only 16% of the participants were found to be hyperglycaemic and 9% were borderline. A small percentage (3%) had high levels of cholesterol, while 26% were considered borderline.

**TABLE 3:** Frequency distribution of body mass index, systolic blood pressure, diastolic blood pressure, cholesterol and glucose.

Variable	Classification†	Cut-off point	*N*	%
BMI (kg/m^2^)	Normal weight	18.5–24. 9	27	36
	Overweight	25–29.9	24	32
	Obesity	≥ 30	24	32
Systolic blood pressure (mmHg)	Normal	< 120	33	44
	Pre-hypertensive	120–139	38	51
	Hypertensive	> 140	4	5
Diastolic blood pressure (mmHg)	Normal	< 80	38	51
	Pre-hypertensive	80–89	26	35
	Hypertensive	≥ 90	11	15
Blood glucose (mmol/L)	Desirable	< 6.1	55	75
	Borderline	6.1–6.9	7	9
	High	> 7.0	12	16
Cholesterol (mmol/L)	Desirable	< 5.2	54	72
	Borderline	5.2–6.2	19	25
	High	> 6.2	2	3

Note: Nineteen per cent of men and 5.7% of women exhibited three or more risk factors. This resulted in an average of 12.4% of participants with three or more risk factors (Table 4).

BMI, body mass index.

† , Classifications from Pescatello (2014).

**TABLE 4:** Risk profiles of automotive workers in different age and gender categories.

Variable	Full group		< 45 years		≥ 45 years		Women		Men	
	*N*	%	*N*	%	*N*	%	*N*	%	*N*	%
No risk	17	22.7	14	25.0	3	25.0	15	27.8	2	9.5
One risk	22	29.3	22	37.9	0	0.0	15	27.8	7	33.3
Two risks	25	33.3	20	34.5	5	41.7	16	29.6	9	42.9
Three risks	7	9.3	5	8.6	2	16.7	5	9.3	2	9.5
Four risks	4	5.3	2	3.4	2	16.7	2	3.7	2	9.5
Five risks	0	0.0	0	0.0	0	0.0	0	0.0	0	0.0

## Discussion

This study examined the cardiovascular and metabolic risk profile of automotive industry workers from a developing country, and the relationship between BMI and various risk factors, which included BP, cholesterol, glucose, age and gender.

In the present study, 66% of the participants were classified as either overweight or obese. These results are similar to the 68% obesity levels reported for Brazilian industrial workers (Cassani et al. 2009). The mean BMI for both the under 45 and over 45 years old groups, as well as the men and women when analysed separately, fell within the overweight category (Tables 1 and 2). This implies that obesity is a common problem and is not limited to certain age or gender categories. Overweight and obesity are considered the biggest risk factors for CVD, stroke, non-insulin-dependent diabetes mellitus, certain cancers and many other illnesses (Akil & Ahmad 2012). A sedentary lifestyle is considered one of the main contributors to the risk factors of CVDs; it is therefore expected that the active work associated with industrial settings should result in reduced rates of CVD risks (Lachman et al. 2018).

However, mechanisation in the workplace, especially in the automotive industry, has limited and, in some instances, removed the physical involvement of workers in their daily tasks. Research has shown that the daily energy expenditure because of work-related physical activity in the United States has decreased by more than 100 kcal during the last 50 years in both men and women (Church et al. 2011). The research further found the reduced energy expenditure to be associated with an increase in mean body weight over the same time (Church et al. 2011). These reduced levels of energy expenditure could be a contributing factor to the obesity problem identified in this study. The globalisation of food production is also considered a major contributor to the increased incidence of obesity in developing countries (Caballero 2007).

The association between age and BMI in this study was found to be positive and significant. According to Hill et al. (2012), the main contributor to low energy throughput that puts people at risk of weight gain is a low level of physical activity.

One of the risk factors identified in this study is high BP. Fifty-six per cent of the participants were found to be hypertensive in terms of their SBP and 50% in terms of their DBP. The levels of hypertension are of concern because only 16% of the workers were older than 45 years. This study found a significant positive correlation between BMI and both SBP and DBP, which concurs with the outcomes of previous studies focussing on the same topic (Dua et al. 2014). Although no causal relationships can be claimed between BMI and BP, the relationship is positive and significant, and the focus of intervention programmes should be on addressing the problems of being overweight and obese. The rapid expansion of the obesity epidemic is happening against the backdrop of continuous decline in the energy expenditure required in the workplace and daily living in general brought about by labour-saving devices. It is recommended that intervention strategies for the reduction of overweight and obesity should include physical activity, as it has been shown to have a positive effect on BP and on achieving a healthy body weight (Chaput et al. 2011; Diaz & Shimbo 2013).

One of the encouraging results emanating from the study is that hypercholesterolemia was observed only in 3% of the participants and high levels of blood glucose in 16%. A large number (72%) had desirable cholesterol levels, while 19% were identified as borderline. The proportion of individuals with desirable blood glucose levels was 75%.

Only 22.7% of the entire population were free from any risk factors related to CVDs. Approximately, 30% of the participants had at least one risk factor, while 33% had two co-existing risk factors. Eleven per cent had three risk factors or more.

Employees in major industries, such as the automotive industry, constitute a large section of the workforce in most industrialised countries. These industries with their extensive in-house resources and health care systems are ideal settings for initiating preventative programmes. Industrial environments further provide ideal captive audiences for the implementation of awareness programmes related to the health risks associated with sedentary lifestyles, including CVDs. It is strongly recommended that industries put systems, facilities and practices in place that will allow and motivate their workers to become physically active.

## Conclusion

It is important to know the health risk factors of a population who spends a considerable amount of their time in a confined setting. This allows health scientists to address their health needs in the most appropriate and effective manner. This study confirms the prevalence of risk factors for CVD among shift workers employed in the automotive industry. The CVD risk factors identified in the current study emphasise the need for health promotion in the workplace especially because the benefits emanating from intervention programmes help to decrease modifiable risk factors.

## Limitations

Because the study extracted data from an existing database, the scope of the study was limited to the information contained therein. However, the depth and width of the data were adequate to describe the CVD and metabolic risk factors of shift workers within the automotive industry.
